# The interoperability between the Spanish version of the International Classification of Diseases and ORPHAcodes: towards better identification of rare diseases

**DOI:** 10.1186/s13023-021-01763-y

**Published:** 2021-03-09

**Authors:** Juan Rico, Luis Javier Echevarría-González de Garibay, María García-López, Sandra Guardiola-Vilarroig, Luis Alberto Maceda-Roldán, Óscar Zurriaga, Clara Cavero-Carbonell

**Affiliations:** 1 Rare Diseases Research Unit, Foundation for the Promotion of Health and Biomedical Research in the Valencia Region, Valencia, Spain; 2 Directorate for Healthcare Planning, Organization and Evaluation, Registries and Health Information Unit, Ministry of Health of the Basque Government, Vitoria-Gasteiz, Spain; 3 Rare Diseases Registry, Public Health Office, Castilla and León Government, Valladolid, Spain; 4 Public Health Regional Health Administration (DG Salud Publica y Adicciones), Generalitat Valenciana, Valencia, Spain; 5 Murcia Region Rare Diseases Information System, Murcia Regional Health Council, Murcia, Spain; 6 Public Health and Preventive Medicine Department, University of Valencia, Valencia, Spain

**Keywords:** Rare diseases, ICD-10-ES, ORPHAcode, Diagnoses, Codification, Healthcare, Public Health

## Abstract

**Background:**

Rare diseases present a wide spectrum of clinical manifestations and severity levels and are often poorly known and underrepresented, making them difficult to classify. Diagnoses are usually coded using the International Classification of Diseases (ICD), with its different versions. In Spain, the ICD-10-ES (stem from the ICD-10-CM–Clinical Modification) is used throughout the National Healthcare System since 2016, indistinctively including rare diseases that often lack a specific code. Orphanet aims to provide high-quality resources on rare diseases. The goal was to interrelate the Orphanet classification with the ICD-10-ES in order to engage a tool to track rare diseases diagnosis and characterize the improvement space for the identification of rare diseases patients in the Spanish Healthcare System.

**Methods:**

5775 disorder level ORPHAcodes were mapped to ICD-10-ES codes by comparing the descriptors associated in both classifications. ORPHAcodes were then clustered based on their assigned ICD-10-ES chapter and the redundancy of each individual ICD-10-ES code was calculated by counting the ORPHAcodes they mapped to. Three groups were established: Group 1 (1 ORPHAcode per ICD-10-ES), Group 2 (between 2–49 ORPHAcodes per ICD-10-ES) and Group 3 (≥ 50 ORPHAcodes per ICD-10-ES).

**Results:**

Equivalences to 1700 ICD-10-ES codes were established for 5664 ORPHAcodes. The ORPHAcodes distribution within the ICD-10-ES showed an aggregation in the “Q” (> 40%), “G” (> 14%), and “E” (12%) chapters. The availability of ICD-10-ES codes to map ORPHAcodes reached its lowest at the “G” and “Q” chapters with less than 0.2 ICD-10-ES codes available per ORPHAcode. Global ICD-10-ES codes redundancy analysis revealed that only 1055 of the equivalences pertain to group 1. Group 2 contained 3358 equivalences with 634 ICD-10-ES codes while 1322 equivalences were group 3 (11 ICD-10-ES). Within ICD-10-ES chapters, “G” and “Q” contained over 30% and 45% of their own equivalences in the highest redundancy level (group 3) respectively, but under 10% one to one equivalences each (group 1).

**Conclusions:**

ICD-10-ES codes have not enough specificity to identify rare diseases. Direct mapping between ICD and ORPHAcodes or the integration of ORPHAcodes at the healthcare system for diagnoses codification would enable better detection and epidemiological analysis of rare diseases.

**Supplementary Information:**

The online version contains supplementary material available at 10.1186/s13023-021-01763-y.

## Background

Rare diseases (RDs) are a group of heterogeneous disorders with highly variable and difficult prognosis whose main common feature is their low prevalence. There is no universal definition of RDs prevalence as rarity thresholds are derived from orphan drug legislation and diagnosis criteria vary throughout the world: in the US, the prevalence for a RD is set under 200,000 cases in the whole country at any time-point, in Japan the prevalence definition is set in 1 or less cases per 2500 individuals, while in the European Union the threshold are 5 people affected for each 10,000 individuals [1–4]. This prevalence threshold has been arbitrarily set in at least 296 definitions to reach a global average of 40 cases every 100,000 people [5, 6]. Moreover, the total number of disorders considered as RDs depends on the country or region, as the definition of RDs and the incidence of the disorders may vary significantly. Overall, an estimated range between 5000 and 8000 RDs is generally accepted and some reports raise this figure to over 10,000 [7].

To keep track of diagnoses, and provide data for statistical analysis, coding systems are used around the globe. The International Classification of Diseases (ICD) endorsed by the World Health Organization (WHO) [8] is widely employed to analyse the burden of particular diseases, or groups of diseases within a healthcare system. However, since the main purpose of this classification is to control and allocate health services expenses and funding, is difficult to make it extensive for research and/or other more specialized purposes. Several alternative classifications have been developed to fulfil the needs that arise under specific circumstances or to treat the data otherwise. For instance, the Systematized Nomenclature of Medicine Clinical Terms (SNOMED-CT) was created to enable health specialists and researchers to share and access clinical knowledge, descriptions, and relationships of diseases [9]. Other classifications are focused on specific groups of diseases such as the ERA-EDTA [10, 11] coding system for renal diseases or the British Paediatric Association variant of the ICD (ICD-10-BPA) [12] developed to classify congenital anomalies. Most of them are focused on the specific traits of diseases or at the hereditary pattern/age of onset but none distinguish them because of their prevalence. That remains a handicap for the identification of RDs, which are scattered in a melange with common diseases within these classifications. However, an effort to cover this gap was undertaken by Orphanet [13], an INSERM derived organization that has developed and maintained a RDs specific classification. The main criterion to include a disorder at the Orphanet classification is its low prevalence (below 1 in 2000 according to the European Union definition of RD), the disease must also be described in at least two different individuals [14]. The Orphanet nomenclature is a multilingual, standardised, controlled medical terminology specific to RDs that includes all clinical entities registered in the Orphanet database. A unique and time-stable numerical identifier called ORPHAcode is randomly attributed by the database upon creation of the entity [14]. The Orphanet nomenclature is organised in a multi-hierarchical and polyparental classification system arranged in three hierarchical levels: Group of disorders, Disorder, and Subtype of a disorder. The disorder level is designated as the main typological level for data sharing and statistical reporting across the European Union [14].

The Spanish National plan for Rare Diseases was established in 2009 under the directions of the European Council (2009/C 151/02) [15], to adapt the national policies to the new framework of RDs. In 2012, the Spanish Rare Diseases Registries Research Network (SpainRDR) was launched to pilot the development of a national and population-based RD registry. The SpainRDR project reported, with a coverage of 80.2% of the Spanish population, 824,399 RD cases for the period 2010–2011 with 26% corresponding to congenital anomalies; 19% endocrine, nutritional and metabolic diseases; 13% blood and blood-forming organs and certain disorders involving the immune mechanism and 10% diseases of the circulatory system among others [16]. The National Registry of Rare Diseases, created by order (1091/2015) in 2015 [17], compiles the diagnoses for RDs by gathering the information from the regional registries. Currently, a subset of RDs is of compulsory notification to the national registry while the rest of them, even if somewhat registered, are kept at hospital or regional level. Each region has a population-based registry that collects metadata from primary information sources including: the hospital discharges database known as CMBD, direct notification by clinicians and other registries including, but not limited to, renal diseases, primary care, drugs, handicapped, congenital anomalies and genetics. The classification systems in which the diagnoses are received from the different information sources are diverse and tables of equivalences are often established to convert, when needed, the input codes to their reference coding system; normally ICD-10 and/or ICD-10-ES.

In Spain, the mortality registry works with the ICD-10 and healthcare services extensively use an adapted version of the clinical modification of the ICD-10 (ICD-10-ES) to code diagnoses since January 2016. The ICD-10-ES is then used at national level to collect morbidity data (through CMBD), which is the main information source for most of the regional RDs registries. Both the ICD-10 and the ICD-10-ES are divided into independent (hardly interrelated) chapters depending on the type of disease or the systems affected. As a result, RDs are blended with common diseases and often share the same codes, making it difficult to discern the information regarding RDs from that unrelated to them. Therefore, the registration of RDs is hindered by the lack of a specific chapter that distinguishes them.

RDs are often the cause of premature death, and are a non-negligible burden not only for patients, but for families and healthcare systems. Point-prevalence analyses have been performed with an estimation of 5.9% of the population being affected worldwide by just two-thirds of the RDs included in the Orphanet database for which the point-prevalence is a good epidemiological indicator [18]. However, the actual figure is likely to be higher as most of the data used for the calculations were based on previous estimations and/or ranges rather than accurate point-prevalence data. Only with thorough and systematic registration of RDs diagnoses, will it be possible to actually infer the prevalence of each RD as well as their global cumulative prevalence. A reliable registration of RDs cases must be in place in order to help to deal with the huge variability both at clinical and administrative level.

The Orphanet database of RDs contains over 6000 disorders excluding groups of disorders and subtypes. Around 72% of them have genetic basis and 70% are of paediatric onset exclusively [18]. During the Joint Action on Rare Diseases, RD-ACTION (2015–2018) [19], the basis to establish equivalences from ICD-10 to ORPHAcodes was settled, as well as the guidelines for the implementation of the latter at European healthcare systems. The RD-CODE project (2019–2021) [20], in which the current study was framed, aims to move this effort forward by implementing ORPHAcodes in 4 European countries (Malta, Romania, Czech Republic and Spain). The goal of the present study was to correlate the 5775 ORPHAcodes resulting from the RD-ACTION to ICD-10-ES codes in order to provide objective data about the codification system in Spain and its efficiency to identify RDs. In addition, we present a tool that sets the basis for RDs regional registries to standardize the way to convert the diagnoses received from their information sources into reliable ORPHAcodes to be transmitted to the national registry.

## Methods

A study to interrelate the ICD-10-ES and ORPHAcode classifications was performed in Spain during 2019–2020 in the frame of the RD-CODE project by a consortium integrated by 6 Regional Rare Diseases Registries covering around 40% of the Spanish population (from the Basque Country, Castile and Leon, Navarre, Catalonia, Murcia and Valencia Region), the Rare Diseases Joint Research Unit FISABIO-UVEG (Foundation for the Promotion of Health and Biomedical Research of Valencia Region—University of Valencia) and the CIBERER (Biomedical Research Networking Centre on Rare Diseases).

### Starting information and reference databases used in the study

The documents “Standard procedure and guide for the coding with ORPHAcodes” and “Specification and implementation manual of the Master file” developed in the frame of the RD-ACTION Joint Action (2015–2018) [21] were used as reference for the proposal and establishment of equivalences between ORPHAcodes and ICD-10-ES codes.

A file named “Master file for statistical reporting with ORPHAcodes” (MF) [21], also developed during the RD-ACTION Joint Action, was the template containing the 5775 disorder level ORPHAcodes targeted in this study, which were originally extracted from Orphadata [22] using the 2018 version of the Orphanet nomenclature. The MF spreadsheet included, when available, the equivalences to ICD-10 (2016 version) codes proposed by Orphanet that were used as indicators to subsequently fulfil the ICD-10-ES codes equivalent to the ORPHAcodes. In addition, two tables (one carried out in the Valencia Region and another one in the Basque Country) containing equivalences from ICD-10-ES codes to a subset of ORPHAcodes previously compiled by their RDs registries, were of use to endorse (or contravene) the equivalences reached in this study.

The procedural document describing the ICD-10 coding rules for rare diseases [23] followed by Orphanet, was also used to understand the process behind the ICD-10 correspondences included at the MF. This document defines the different ways an ICD-10 code can be attributed to an ORPHAcode and the relationship between them. These pre-established rules were the basis to support the criteria later adopted in this study.

### Establishment of the equivalence proposal workflow(s)

The ORPHAcodes listed in the MF and its associated data were checked out at the Orphanet database of RDs [24] to corroborate their validity. Thereafter, the main descriptors (also known as “preferred term”), their synonyms and the ICD-10 codes linked to the ORPHAcodes (if any) were searched at the second edition of the ICD-10-ES classification (2018) allocated at the eCIE-Maps server from the Ministry of Health, Consumer Affairs and Social Welfare (MSCBS) [25] to select the most accurate ICD-10-ES code for each ORPHAcode. The ICD-10-ES codes reached by this method were then compared to those proposed in the previous databases developed in the Valencia Region and in the Basque Country.

The criteria to choose the codes among the possible options offered by the ICD-10-ES were discussed during a face-to-face meeting of the consortium, with the participation of the MSCBS. Representatives from the departments of codification of some hospitals were also present to discuss their current and forecasted strategies to include RDs diagnoses within their hospital databases. The document summarizing the agreements reached was then written and formally accepted by the consortium, establishing thenceforth the standard workflow for the proposal of equivalences between ICD-10-ES codes and ORPHAcodes.

After the setup of the standard workflow, a number of non-canonical equivalences seemed to escape the boundaries established in it. A survey was then generated to collect the different circumstances arisen while trying to establish equivalences based on the criteria agreed in the first place. The survey, including at least two different approaches to establish the equivalences under the newly found circumstances, was circulated among the consortium. The answers registered were then studied and an extended workflow was written and approved.

From this point onwards, the standard workflow was applied to all the equivalences but resorting to the extended workflow every time a non-canonical equivalence was found. If, by any means, one or more equivalences were yet difficult to define by the criteria described in them, those were gathered and distributed at the end of each month for individual analysis and assessment by the mapping team, integrated by FISABIO and the RDs registries that agreed to perform regular surveillance of the proposal of equivalences (Basque Country, Castile and Leon, Murcia and Valencia Region). Once the first round of proposals for the whole MF was completed, the equivalences that remained unsolved were aggregated in order to be revised once again by the mapping team. The ICD-10-ES codes corresponding to the ORPHAcodes sent for revision, either monthly or at the end of the first equivalences proposal attempt, were only accepted when at least 3 out of the 5 members of the mapping team agreed upon the same correspondence. The mapping team was actively involved in the proposal of equivalences and in their review, with FISABIO acting both as member and coordinator.

### Analysis of ORPHAcodes distribution among the ICD-10-ES chapters

The equivalences established from ORPHAcodes to ICD-10-ES codes were treated with a Microsoft Excel pivot table in order to arrange and divide them based on the ICD-10-ES chapter they belong to. The ORPHAcodes mapped to each ICD-10-ES chapter were counted and its relative abundance among the total number of ORPHAcodes calculated with the following formula:

$$Relative\,chapter\,weight\,(\% ) = \frac{Number\,of\,ORPHAcodes\,mapped\,to\,the\,ICD\,chapter}{{Total\,number\,of\,ORPHAcodes\,mapped\,in\,the\,study}} \times 100$$

The relative amount of ICD-10-ES codes from each chapter used to establish equivalences in this study was also calculated with the formula:

$$Relative\,amount\,of\,ICD\,codes\,(\% ) = \frac{Number\,of\,ICD\,codes\,used\,from\,the\,chapter}{{Total\,number\,of\,ICD\,codes\,used\,in\,the\,study}} \times 100$$

### Calculating the ratio of ICD-10-ES codes available per ORPHAcode

The capability of the ICD-10-ES classification and its individual chapters to cope with the number of ORPHAcodes mapped to them was first explored by calculating the ratio of ICD-10-ES codes (available) per ORPHAcode. To do so, the data generated to do the previous calculations of relative abundance were used as the variables of the following formula:

$$Ratio\,of\,ICD\,codes\,per\,ORPHAcode = \frac{Number\,of\,ICD\,codes}{{Number\,of\,corresponding\,ORPHAcodes}}$$

### Analysis of ICD-10-ES codes redundancy: global, per chapter, section and code

In order to calculate the redundancy of the ICD-10-ES equivalences, the Microsoft Excel pivot table counting the use of each ICD-10-ES code was filtered. First of all, ORPHAcodes were classified into three groups according to the redundancy of their matching ICD-10-ES codes. Group 1 containing the ORPHAcodes whose ICD-10-ES code was just assigned once (to that ORPHAcode only), group 2 containing the ORPHAcodes whose ICD-10-ES code was assigned to more than one ORPHAcode and up to 49 different ORPHAcodes and group 3 containing the ORPHAcodes whose ICD-10-ES code was assigned to 50 or more different ORPHAcodes. Once the groups were delimited, the calculation of the redundancy levels was done as follows:

$$ORPHAcodes\,per\,group\,(\% ) = \frac{Number\,of\,ORPHAcodes\,of\,the\,group}{{Total\,number\,of\,ORPHAcodes \left( {global, chapter, section} \right)}} \times 100$$

### Quantification of ORPHAcodes per ICD-10-ES: per section and code

The ORPHAcodes corresponding to chapters with high-redundancy ICD-10-ES codes (group 3) were gathered based on the section of the chapter they mapped to. The ORPHAcodes corresponding to each section were counted to decipher the ORPHAcodes distribution within these chapters.

$$Number\,of\,ORPHAcodes\,of\,section\,n = ORPHAcodes\,mapped\,to\,\left( {code_{n0} + code_{n1} + code_{nn} } \right)$$

The ORPHAcodes corresponding to each individual ICD-10-ES code were counted with the Microsoft Excel pivot table in order to determine which ICD-10-ES codes were the most redundant within the highest redundancy chapters and sections.

## Results

**Fig. 1:**
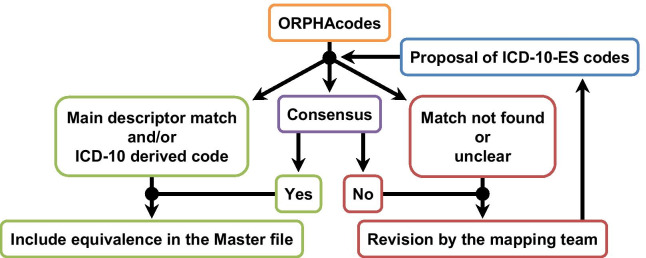
Standard workflow for the proposal of equivalences between ORPHAcodes and ICD-10-ES codes

Equivalences to ICD-10-ES were attempted for the ORPHAcodes of the 5775 disorder level entities from the Orphanet database of RDs included at the MF, following the criteria agreed (Fig. 1) by the consortium.

To illustrate the different situations and how they were solved according to these criteria, six representative examples have been selected and are described below. In the standard workflow, the first criterion was to prioritise codes with matching descriptors in both classifications, ICD-10-ES and ORPHAcode (example 1).

### Example 1

ORPHAcode: 111 → Descriptor: Barth syndrome

ICD-10 code proposed by Orphanet: E71.1 → Descriptor: Other disorders of branched-chain amino acid metabolism

ICD-10-ES code proposed by this study: E78.71 → Descriptor: Barth syndrome

In the cases where matching descriptors were not found, the second criterion applied was that the ICD-10-ES code derived from the ICD-10 code proposed by Orphanet would be chosen if available (example 2).

### Example 2

ORPHAcode: 2045 → Descriptor: FLOTCH syndrome

ICD-10 code proposed by Orphanet: L60.8 → Descriptor: Other disorders of nails

ICD-10-ES code proposed by this study: L60.8 → Descriptor: Other disorders of nails

When those criteria could not be fulfilled, the ORPHAcodes were submitted along with their potential ICD-10-ES codes to the mapping team in order to agree upon the best equivalence to ICD-10-ES.

**Fig. 2:**
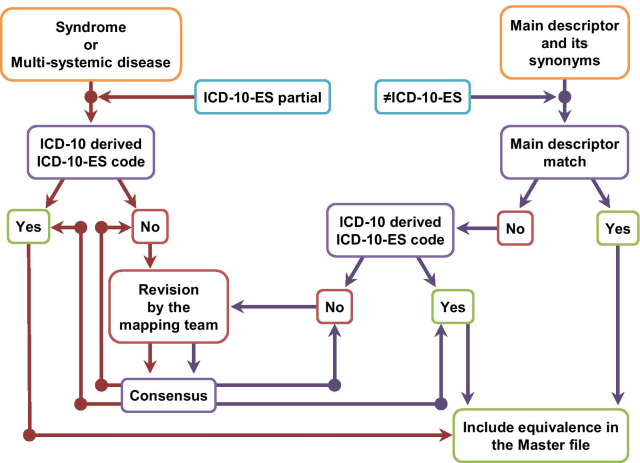
Extended workflow for the proposal of equivalences between ORPHAcodes and ICD-10-ES codes under specific circumstances

When more than one ICD-10-ES code seemed a suitable correspondence to an ORPHAcode, the extended workflow was activated (Fig. 2).

In the cases were a multi-systemic disease or syndrome was classified under a disorder level ORPHAcode, but there was no ICD-10-ES code fully matching the disorder features, the criterion was to prioritise the ICD-10-ES code derived from the ICD-10 code proposed by Orphanet (example 3).

### Example 3

ORPHAcode: 2585 → Descriptor: Ataxia-pancytopenia syndrome

ICD-10 code proposed by Orphanet: D61.0 → Descriptor: Constitutional aplastic anemia

ICD-10-ES code proposed by this study: D61.09 → Descriptor: Other constitutional aplasias

Complementary (not selected) ICD-10-ES code: G11.8 → Descriptor: Other hereditary ataxias

Secondly, in the cases where the main descriptor and one or more of its synonyms listed at Orphanet for one ORPHAcode match different ICD-10-ES codes, the criteria were to prioritise the ICD-10-ES code which descriptor matches the main descriptor associated to the ORPHAcode or in its absence, the one that derives from the ICD-10 code proposed by Orphanet (example 4).

### Example 4

ORPHAcode: 86879 → Descriptor: Extranodal nasal NK/T-cell lymphoma

Synonym: Lethal midline granuloma

ICD-10 code proposed by Orphanet: C86.0 → Descriptor: Extranodal NK/T-cell lymphoma, nasal type

ICD-10-ES code proposed by this study: C86.0 → Descriptor: Extranodal NK/T-cell lymphoma, nasal type

Alternative (not selected) ICD-10-ES code: M31.2 → Descriptor: Lethal midline granuloma

Anytime these criteria failed to produce a reasonable equivalence, the third step was to submit these ORPHAcodes along with their potential (if any) ICD-10-ES codes to the mapping team.

When multiple ICD-10-ES codes were correlated to the same ORPHAcode, they were clustered under a generic code constructed with their common “prefix” followed by a plus “ + ” symbol. This was made to avoid overstating univocal (group 1) equivalences when several ICD-10-ES codes were matching the same ORPHAcode (example 5).

### Example 5

ORPHAcode: 1163 → Descriptor: Aspergillosis

ICD-10 code proposed by Orphanet: B44 +  → Descriptor: Aspergillosis

ICD-10-ES code proposed by this study (for data treatment): B44 +  → Descriptor: Aspergillosis

Equivalent ICD-10-ES code(s) (for use at registries): B44.0; B44.1; B44.2; B44.7; B44.8 (B44.81; B44.89); B44.9

In addition, when an ORPHAcode equivalent ICD-10-ES code(s) or a subset of them was included within the multiple ICD-10-ES equivalences already clustered for another ORPHAcode(s), the ICD-10-ES code(s) of these new ORPHAcode(s) were also converted to the same condensed ICD-10-ES {common prefix followed by “ + ”} code (example 6).

### Example 6

ORPHAcode: 1164 → Descriptor: Allergic bronchopulmonary aspergillosis

ICD-10 code proposed by Orphanet: B44.1 +  → Descriptor: Other pulmonary aspergillosis

ICD-10-ES code proposed by this study (for data treatment): B44 +  → Descriptor: Aspergillosis

Equivalent ICD-10-ES code(s) (for use at registries): B44.81 → Descriptor: Allergic bronchopulmonary aspergillosis

Examples 5 and 6 illustrate different situations in which ICD-10-ES code(s) were converted to their condensed form for data treatment. In the first, the ORPHAcode 1163 had equivalences to 8 different ICD-10-ES codes that share the root “B44”. The other example shows ORPHAcode 1164, which had the ICD-10-ES code B44.81 as its sole equivalence. Nonetheless, B44.81 is common to both, 1163 and 1164 ORPHAcodes, making necessary to homogenize the equivalence in order to not count this equivalence as unique. By applying this strategy, we minimize the bias resulting from multiple ICD-10-ES equivalences to one ORPHAcode over the calculations. A total of 834 equivalences were adjusted by these means, and around 70% of them were similar to that exposed in example 5.

After all this process, 4987 ORPHAcodes were mapped to ICD-10-ES by applying the standard and extended workflows meanwhile equivalences for 677 were solved only after revision by the mapping team, reaching over 98% of the target ORPHAcodes. The remaining 111 ORPHAcodes (< 2%) lacked of ICD-10-ES code equivalence after exhausting the resolution framework applied in this study, including 94 of the initial 484 without ICD-10 code proposed by Orphanet.

1700 different ICD-10-ES codes were selected to produce 5735 equivalences for these 5664 ORPHAcodes successfully correlated between coding systems. This mismatch between the number of ORPHAcodes and the number of equivalences is due to the fact that 65 ORPHAcodes kept more than one corresponding ICD-10-ES code (making a total of 136 equivalences), because they did not share a common prefix. Moreover, 33 out of these 65 ORPHAcodes established equivalences to more than one ICD-10-ES chapter (See Additional file 1).

### Distribution of ORPHAcodes among the ICD-10-ES chapters

**Fig. 3:**
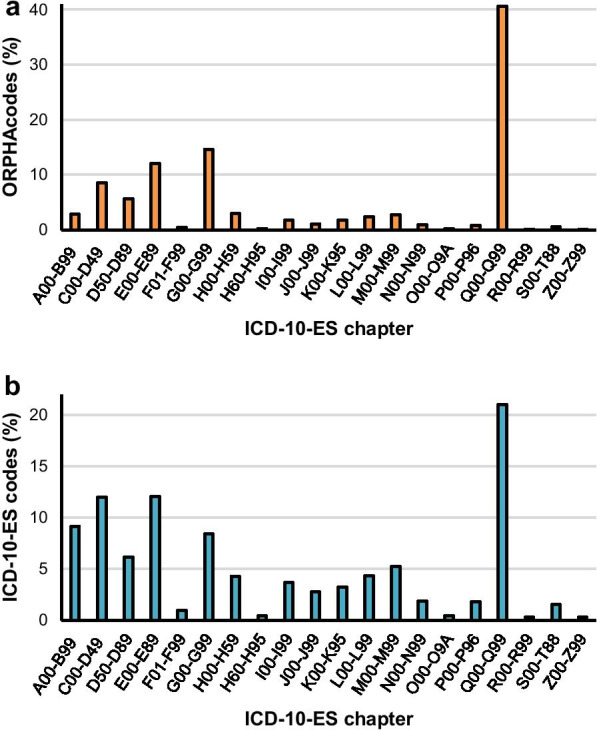
Distribution of codes analysed in this study. **a** Relative number of ORPHAcodes assigned to each ICD-10-ES chapter. **b** Relative amount of ICD-10-ES codes used to establish equivalences to ORPHAcodes per ICD-10-ES chapter

The 5664 ORPHAcodes mapped to ICD-10-ES codes were grouped depending on the ICD-10-ES chapter to whom their assigned ICD-10-ES codes belonged. Twenty out of twenty-one ICD-10-ES chapters (See Additional file 2) got at least one matching ORPHAcode and the distribution of the equivalences was not proportional among them. Three ICD-10-ES chapters gathered over two thirds of the equivalences: “Endocrine, nutritional and metabolic diseases” (E00-E89) with around 12%, “Diseases of the nervous system” (G00-G99) with over 14% and “Congenital malformations, deformations and chromosomal abnormalities” (Q00-Q99) with over 40% (Fig. 3a). The use of ICD-10-ES codes, although followed a similar trend, showed a reduction in the relative weight of these groups. Almost halving in the cases of chapter G with just over 8% and chapter Q with only 21% of the total ICD-10-ES codes used in this study (Fig. 3b).

### Global overview of ICD-10-ES codes availability to map ORPHAcodes

**Fig. 4:**
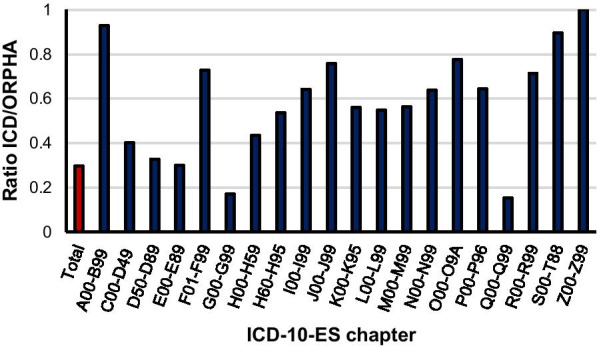
Ratio of ICD-10-ES codes available per ORPHAcode assigned. Red bar (average for the total ICD-10-ES codes/ORPHAcodes), blue bars (ratio per ICD-10-ES chapter)

Once stated that the overall distribution of ORPHAcodes per ICD-10-ES chapter was uneven, the next step was to assess the ratio of available ICD-10-ES codes per ORPHAcode, both globally and within chapters. Data analysis showed dissimilar results among the chapters, 7 chapters showed over 0.7 ICD-10-ES/ORPHAcode (“Certain infectious and parasitic diseases” (A00-B99): 0.93; “Mental, behavioral and neurodevelopmental disorders” (F01-F99): 0.73; “Diseases of the respiratory system” (J00-J99): 0.76; “Pregnancy, childbirth and the puerperium” (O00-O09A): 0.78; “Symptoms, signs and abnormal clinical and laboratory findings, not elsewhere classified” (R00-R99): 0.71; “Injury, poisoning and certain other consequences of external causes” (S00-T88): 0.90 and “Factors influencing health status and contact with health services” (Z00-Z99): 1.00) and 2 chapters showed below 0.2 ICD-10-ES/ORPHAcode (“Diseases of the nervous system” (G00-G99): 0.17 and “Congenital malformations, deformations and chromosomal abnormalities” (Q00-Q99): 0.15) (Fig. 4). Remarkably, the seven chapters with the highest ratio comprise altogether around 5% of the total ORPHAcodes and A00-B99 compiles around 3% on its own. On the other hand, the two chapters with the lowest ratios (G and Q) represent more than half of the total ORPHAcodes included in this study with 4 out of 10 being equivalent to codes from the Q chapter. All these circumstances made the average ratio to fall to around 0.3 ICD-10-ES codes available per ORPHAcode (Fig. 4).

### Global and chapter level analysis of ICD-10-ES codes redundancy

**Fig. 5:**
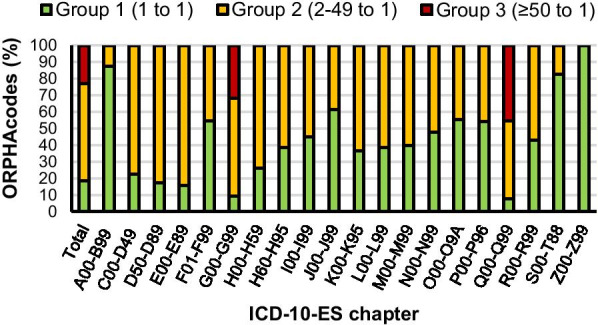
Distribution of ORPHAcodes based on the redundancy of their matching ICD-10-ES code

So far there was an uneven distribution of ORPHAcodes among ICD-10-ES chapters and, moreover not all of the chapters had (enough) specific codes to match the ORPHAcodes assigned to them. Therefore, to deepen in the analyses, the patterns of the equivalences established to individual ICD-10-ES codes were studied. The results showed that ≈18% (1055) of the total equivalences belonged to group 1 (1 ORPHAcode to 1 ICD-10-ES) while ≈23% (1322) fell into group 3 (≥ 50 ORPHAcodes to 1 ICD-10-ES), leaving ≈59% (3358) in group 2 (2 to 49 ORPHAcodes to 1 ICD-10-ES). The in-chapter distribution was studied to further characterize the capabilities of each ICD-10-ES chapter to absorb its share of ORPHAcodes. Yet again, chapters G (9.1%) and Q (7.6%) showed the lowest group 1 ORPHAcodes relative content and were also the only ones containing group 3 ORPHAcodes (31.7% and 45.6% respectively) (Fig. 5).

### Section level assessment of the G chapter codes of the ICD-10-ES

**Fig. 6:**
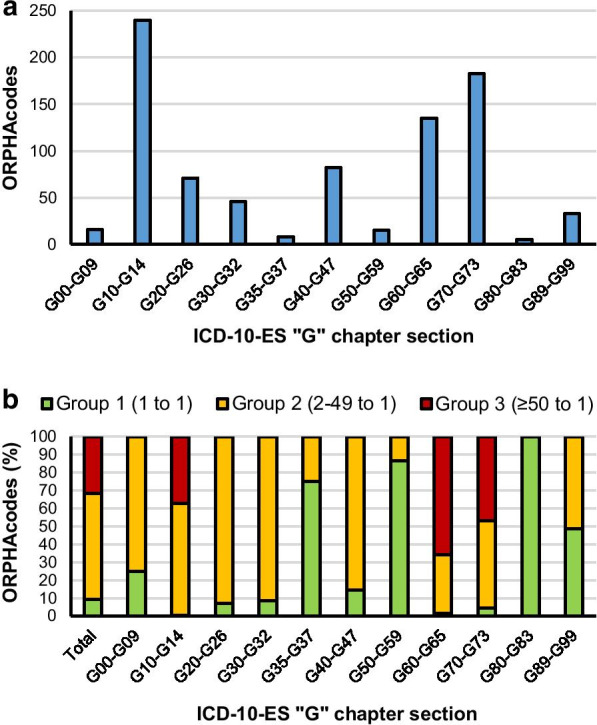
Distribution of ORPHAcodes within the G00-G99 chapter of the ICD-10-ES. **a** Number of ORPHAcodes per section. **b** Distribution of ORPHAcodes based on the redundancy of their matching ICD-10-ES code within the G00-G99 chapter and its sections

Taking a deeper look at the G chapter of the ICD-10-ES, the distribution of ORPHAcodes into the different sections was assessed. Three of its eleven sections contained more than half of the 834 ORPHAcodes assigned to this chapter (“G10-G14—Systemic atrophies primarily affecting the central nervous system”: 240 ORPHAcodes; “G60-G65—Polyneuropathies and other disorders of the peripheral nervous system”: 135 ORPHAcodes; and “G70-G73—Diseases of myoneuronal junction and muscle”: 183 ORPHAcodes) (Fig. 6a). Group 3 ORPHAcodes were also limited to these 3 sections. Between one and two of every three ORPHAcodes assigned to each of them (G10-G14: 37.1%; G60-G65: 65.9% and G70-G73: 47.0%) fell into the group 3 of ICD-10-ES codes redundancy (Fig. 6b).

Regarding specific ICD-10-ES codes, “G11.4—Hereditary spastic paraplegia”: 89 ORPHAcodes; “G60.0—Hereditary motor and sensory neuropathy”: 89 ORPHAcodes; and “G71 + (in particular G71.0—Muscular dystrophy- and G71.8—Other primary disorders of muscles-)”: 86 ORPHAcodes, were the only ones with a redundancy of at least 50 ORPHAcodes each. Therefore, the 264 ORPHAcodes belonging to group 3 in the G chapter can be traced down to these 3 ICD-10-ES codes (See Additional file 3).

### Section level assessment of the Q chapter codes of the ICD-10-ES

**Fig. 7:**
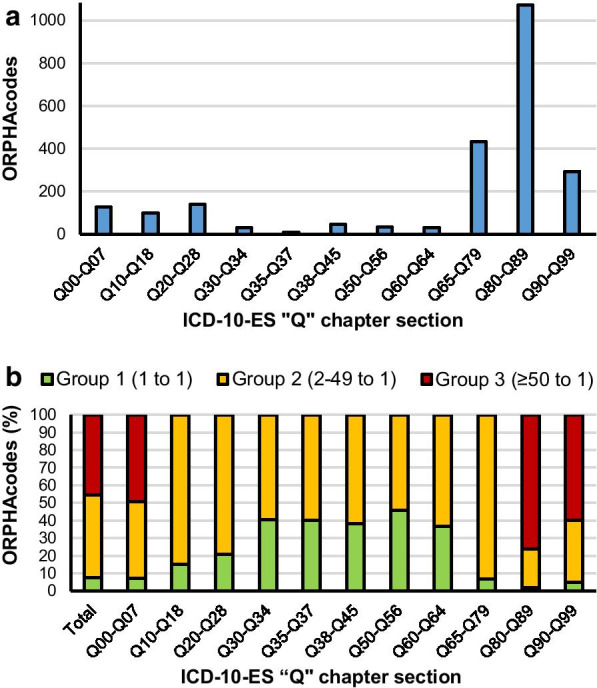
Distribution of ORPHAcodes within the Q00-Q99 chapter of the ICD-10-ES. **a** Number of ORPHAcodes per section. **b** Distribution of ORPHAcodes based on the redundancy of their matching ICD-10-ES code within the Q00-Q99 chapter and its sections

Similarly to what happened with the G chapter of the ICD-10-ES, the distribution of ORPHAcodes into the different sections of the Q chapter was studied. Three of its eleven sections contained more than half of the 2320 ORPHAcodes assigned to this chapter (“Q65-Q79—Congenital malformations and deformations of the musculoskeletal system: 434 ORPHAcodes; “Q80-Q89—Other congenital malformations”: 1072 ORPHAcodes and “Q90-Q99—Chromosomal abnormalities, not elsewhere classified”: 294 ORPHAcodes) (Fig. 7a). Group 3 ORPHAcodes were also limited to 3 sections, but in this occasion a much smaller section (Q00-Q07—Congenital malformations of the nervous system) with just 128 ORPHAcodes assigned had 49.2% of them into group 3. Up to 3 out of 4 of the ORPHAcodes assigned to the other 2 sections (Q80-Q89: 76.4% and Q90-Q99: 59.9%) fell into the group 3 of ICD-10-ES codes redundancy (Fig. 7b).

Once again, when specific ICD-10-ES codes were analysed, just a few of them: “Q04.3—Other reduction deformities of brain”: 63 ORPHAcodes; “Q82.8—Other specified congenital malformations of the skin”: 96 ORPHAcodes; “Q87.0—Congenital malformation syndromes predominantly affecting facial appearance”: 127 ORPHAcodes; “Q87.1—Congenital malformation syndromes predominantly associated with short stature”: 68 ORPHAcodes; “Q87.2—Congenital malformation syndromes predominantly involving limbs”: 57 ORPHAcodes; “Q87.89—Other specified congenital malformation syndromes, not elsewhere classified”: 471 ORPHAcodes; “Q92.2—Partial trisomy”: 68 ORPHAcodes; and “Q93.5—Other deletions of part of a chromosome”: 108 ORPHAcodes showed redundancy of over 50 ORPHAcodes each. Therefore, the 1058 ORPHAcodes belonging to group 3 in the Q chapter can be traced down to these 8 ICD-10-ES codes (See Additional file 4).

## Discussion

During the present work, for the first time, a number of harmonized criteria for the mapping between ORPHAcode and ICD-10-ES were set up and adopted by the RDs registries of six Spanish regions: the Basque Country, Castile and Leon, Catalonia, Navarre, Murcia and Valencia. The implementation of these criteria engages these registries to produce homogeneous and comparable information about RDs at least in terms of ORPHAcodes. Despite this homogenization, finding equivalent ICD-10-ES codes for the ORPHAcodes was not always straightforward because of the scarcity of matching descriptors between both classifications. Indeed, a huge proportion of the disorders studied were not found in the ICD-10-ES list of diagnoses as individual entities, thus had to be mapped to generic ICD-10-ES codes that enclose that kind of disorder. Yet, by using the workflows designed to overcome these drawbacks, an ICD-10-ES mapping was assigned to a significant number of the ORPHAcodes contained in the MF (98%). This effort promises to set the basis for routine implementation of ORPHAcodes in Spanish RDs registries but is nonetheless constrained to the original MF, which is somehow outdated. Indeed, around 2% of the ORPHAcodes listed on it was already obsolete or deprecated by the time the study was finished on June 2020, making the continuous revision of this dataset a need for further real-time analysis of the equivalences. Periodic surveillance of the Orphanet nomenclature and classification is suggested in order to track the relevant changes (e.g. inactive or newly added ORPHAcodes) as well as potential discrepancies on ICD-10 correspondences. Some efforts to address this issue are already ongoing to shape the most suitable strategy.

The MF containing the equivalences between ICD-10-ES and ORPHAcode, along with the pivotal role played by the ICD-10-ES at the RDs regional registries, will facilitate the conversion of the diagnoses received from the multiple information sources to ORPHAcodes, as correlations among input codes and ICD-10-ES were already in use by their systems. Thorough implementation of the ICD-10-ES to ORPHAcodes equivalences would further allow the communication of RDs between Spain and other European countries like France, Italy or Germany which have already reached an advanced stage in the implementation of ORPHAcodes and also with the Czech Republic, Malta and Romania which are also advancing towards their implementation within the RD-CODE project. The implementation of ORPHAcodes as a common language for the codification of RDs around Europe has even the potential to eventually trigger the establishment of a European registry for RDs.

Regarding the analysis of the mapping results, it is quite remarkable the aggregation observed especially at the “Q”, but also at the “G” and “E” chapters of the ICD-10-ES. Not surprising though considering that according to Nguengang Wakap et al. [18] approximately 72% of the RDs allocated at Orphanet have a genetic base and almost 70% are of paediatric onset only. Should this be proven true, reinforcement of the research and healthcare activities regarding RDs in these fields must be encouraged. The uneven distribution of ORPHAcodes resulted in ICD-10-ES codes shortage especially in the “Q” and “G” chapters (less than 0.2 ICD-10-ES codes per ORPHAcode) which, as a consequence, accumulated the most generic and the least specific equivalences. Apropos of the previous, just eleven ICD-10-ES codes compile over 1300 equivalences to ORPHAcodes. The disorders coded by them would be hardly identified in a pure ICD-10-ES based system, thus requiring extra validation steps and creating an additional burden to the registries and an undesired delay over the identification of the disorders within this group. Furthermore, the redundancy of ICD-10-ES codes is expected to be even higher because these with low specificity are likely to bear also equivalences to non-rare disorders which have not been studied here and were not included in the calculations. Therefore, the retrieval of these codes from information sources would not necessarily imply the occurrence of a RD and non-rare disorders would need to be filtered first.

The “Q” chapter dedicated to “Congenital malformations, deformations and chromosomal abnormalities”, was the less adapted to absorb the rare disorders mapped to it with over 1000 highly unspecific equivalences. As mentioned before, another classification derived from ICD-10 (ICD-10-BPA) is in use for the codification of global birth defects. This classification, which is intensively used by the European network of population-based registries for the epidemiological surveillance of congenital anomalies (EUROCAT) [26], might help to partially patch the limitations of the “Q” chapter of the ICD-10-ES in the meantime the ORPHAcodes can be fully implemented in the national healthcare system.

Subsequent revisions of the ICD, like the eleventh revision (ICD-11) are meant to have broader and better structured representation of RDs and to include links to other classifications included its predecessor ICD-10. However, an independent chapter for multi-systemic diseases was dismissed for the time-being [27]. Moreover, the ICD-11 is still in the process of translation and adaptation for its implementation by member states and, according to WHO, is not expected to be used before January 2022 [28].

Assuming the successful implementation of the equivalences between ICD-10-ES and ORPHAcodes and its subsequent versions and/or updates within the Spanish Healthcare System, the impact of this effort over the absolute number of patients identified remains unclear. This is so because the majority of the RDs recorded at Orphanet have a point prevalence below 1 per million [18] and so are included among the so-called ultra-rare disorders. However, patients with RDs do often require medical attention above the average (e.g. in terms of tests and follow-up) thus each case correctly registered may have a significant impact on the health services and the patients’ and families’ life quality. Besides, undiagnosed patients would be yet out of reach with this strategy as there is not a reliable system to keep registry of the patients in this situation and/or track changes in their diagnosis. The Spanish Undiagnosed Rare Diseases Program (SpainUDP) [29] and, at the European level, the Solve-RD project [30] aim to tackle the issue of undiagnosed patients, and hopefully, the efforts being held in collaboration between the RD-CODE, SpainUDP and Solve-RD among others will also lead to improvements in the registration of these patients.

In summary, despite potential limitations, to reach a more accurate registry of RDs, the coding system(s) used to classify the diagnoses at primary care, hospital discharge and other healthcare activities should be updated to include as many specific codes for RDs as needed. The update of the ICD-10-ES chapters that enclose the most of the ORPHAcodes should be encouraged. Otherwise, multiple coding systems, including ORPHAcode, should be of regular use in order to speed up the identification process and allow calculating the prevalence of each RD within different populations. Only if all RD cases are correctly recorded will we be able to optimise resources and maximise the impact, improving not only the patients’ healthcare and treatment, but also bringing up better tools for the families and researchers.

## Conclusions

This study sets the basis for the systematic assignment of ORPHAcodes to the diagnoses of RDs collected at regional and national level Spanish registries. On the other hand, the results obtained when the equivalences established between ICD-10-ES and ORPHAcodes were characterized in terms of specificity and redundancy highlight the poor interoperability between the system for diagnoses track in Spain (ICD-10-ES) and the RDs specific classification created by Orphanet (ORPHAcode).

The fact that most ORPHAcodes were included in a few chapters of the ICD-10-ES indicates that, for unknown reasons, there is a trend in the dataset towards certain types of disorders, genetic inheritance, or age. Reliable identification systems must be implemented for all RDs and this study showcases that a huge amount of the effort should be oriented towards the codification of congenital anomalies and nervous system diseases.

## Supplementary Information

**Additional file 1: Table S1**. List of ORPHAcodes that match ICD-10-ES codes in different chapters

**Additional file 2: Table S2**. List of chapters of the Spanish ICD-10-CM (ICD-10-ES), range of codes and number of equivalences to ORPHAcodes

**Additional file 3: Fig. S1**. Distribution of ORPHAcodes per ICD-10-ES code of the G00-G99 chapter of the ICD-10-ES

**Additional file 4: Fig. S2**. Distribution of ORPHAcodes per ICD-10-ES code of the Q00-Q99 chapter of the ICD-10-ES

## Data Availability

The datasets supporting the conclusions of this article are available in the e-CIE-Maps server, https://eciemaps.mscbs.gob.es/ecieMaps/browser/index_10_mc_old.html, The Orphanet database of rare diseases, https://www.orpha.net/consor/cgi-bin/Disease.php?lng=ES, and the RD-Action website, http://www.rd-action.eu/leaflet-and-documents/. The updated Master file with the correspondences to ICD-10-ES will be an outcome of the RD-CODE project and will be available through its website http://www.rd-code.eu/about-us/ at its closure or on demand.

## Abbreviations

CIBERER: Biomedical Research Networking Centre on Rare Diseases
FISABIO-UVEG: Foundation for the Promotion of Health and Biomedical Research of Valencia Region: University of Valencia
ICD-10: International Classification of Diseases, tenth revision
ICD-10-ES: International Classification of Diseases, tenth revision (Spanish edition)
ICD-11: International Classification of Diseases, eleventh revision
MF: Master file for statistical reporting with ORPHAcodes
MSCBS: Ministry of Health, Consumer Affairs and Social Welfare
RD: Rare disease
WHO: World Health Organization
