# Supplementary material for: The interoperability between the Spanish version of the International Classification of Diseases and ORPHAcodes: towards better identification of rare diseases
Source: Orphanet J Rare Dis. 2021 Mar 9;16:121. doi: 10.1186/s13023-021-01763-y (PMC7941896; doi:10.1186/s13023-021-01763-y)
Supplement: Supplementary file 1 — Additional file 1: Table S1. List of ORPHAcodes that match ICD-10-ES codes in different chapters [file 13023_2021_1763_MOESM1_ESM.pdf]

Supplementary Table 1. List of ORPHAcodes* that match ICD-10-ES codes in different chapters.

ORPHAcode	Main descriptor	ICD-10-ES chapter	
298	Mitochondrial neurogastrointestinal encephalomyopathy	E00-E89	G00-G99
340	Hemorrhagic fever-renal syndrome	A00-B99	N00-N99
567	22q11.2 deletion syndrome	D50-D89	Q00-Q99
723	Pneumocystosis	A00-B99	J00-J99
779	Reynolds syndrome	K00-K95	L00-L99
800	Schwartz-Jampel syndrome	G00-G99	Q00-Q99
913	Zollinger-Ellison syndrome	C00-D49	E00-E89
1164	Allergic bronchopulmonary aspergillosis	A00-B99	J00-J99
1930	Herpes simplex virus encephalitis	A00-B99	G00-G99
2965	Prolactinoma	C00-D49	E00-E89
3452	Whipple disease	K00-K95	M00-M99
49804	Lichen amyloidosis	E00-E89	L00-L99
83452	Complex regional pain syndrome	G00-G99	M00-M99
83616	Rubella panencephalitis	A00-B99	G00-G99
85447	ATTRV30M amyloidosis	E00-E89	G00-G99
85451	ATTRV122I amyloidosis	E00-E89	I00-I99
85453	X-linked reticulate pigmentary disorder	E00-E89	L00-L99
85458	Hereditary cerebral hemorrhage with amyloidosis	E00-E89	I00-I99
90078	Invasive infections due to vancomycin-resistant enterococci	A00-B99	Z00-Z99
96253	Cushing disease	C00-D49	E00-E89
99930	Secondary pulmonary hemosiderosis	E00-E89	J00-J99
99931	Idiopathic pulmonary hemosiderosis	E00-E89	J00-J99
137810	Nodular cutaneous amyloidosis	E00-E89	L00-L99
137814	Macular amyloidosis	E00-E89	L00-L99
220465	Laron syndrome with immunodeficiency	D50-D89	E00-E89
220493	Joubert syndrome with ocular defect	H00-H59	Q00-Q99
231401	Alpha-thalassemia-myelodysplastic syndrome	C00-D49	D50-D89
279894	Toxic maculopathy due to antimalarial drugs	H00-H59	S00-T88
319247	Hantavirus pulmonary syndrome	A00-B99	J00-J99
319635	Amyloidosis cutis dyschromia	E00-E89	L00-L99
353220	Familial primary localized cutaneous amyloidosis	E00-E89	L00-L99
439254	ITM2B amyloidosis	E00-E89	I00-I99
439854	Fatal congenital hypertrophic cardiomyopathy due to glycogen storage	E00-E89	G00-G99

*The ORPHAcodes were originally extracted from the 2018 version of the Orphanet nomenclature.
