# Supplementary material for: The interoperability between the Spanish version of the International Classification of Diseases and ORPHAcodes: towards better identification of rare diseases
Source: Orphanet J Rare Dis. 2021 Mar 9;16:121. doi: 10.1186/s13023-021-01763-y (PMC7941896; doi:10.1186/s13023-021-01763-y)
Supplement: Supplementary file 2 — Additional file 2: Table S2. List of chapters of the Spanish ICD-10-CM (ICD-10-ES), range of codes and number of equivalences to ORPHAcodes [file 13023_2021_1763_MOESM2_ESM.pdf]

Supplementary Table 2. List of chapters of the Spanish ICD-10-CM (ICD-10-ES), range of codes and number of equivalences to ORPHAcodes*.

ICD-10-ES chapter name	Range	Equivalences
Chapter 01. Certain infectious and parasitic diseases	A00-B99	168
Chapter 02. Neoplasms	C00-D49	507
Chapter 03. Diseases of the blood and blood-forming organs and certain disorders involving the immune mechanism	D50-D89	320
Chapter 04. Endocrine, nutritional and metabolic diseases	E00-E89	684
Chapter 05. Mental, behavioral and neurodevelopmental disorders	F01-F99	22
Chapter 06. Diseases of the nervous system	G00-G99	834
Chapter 07. Diseases of the eye and adnexa	H00-H59	168
Chapter 08. Diseases of the ear and mastoid process	H60-H95	13
Chapter 09. Diseases of the circulatory system	I00-I99	98
Chapter 10. Diseases of the respiratory system	J00-J99	62
Chapter 11. Diseases of the digestive system	K00-K95	98
Chapter 12. Diseases of the skin and subcutaneous tissue	L00-L99	135
Chapter 13. Diseases of the musculoskeletal system and connective tissue	M00-M99	158
Chapter 14. Diseases of the genitourinary system	N00-N99	50
Chapter 15. Pregnancy, childbirth and the puerperium	O00-O9A	9
Chapter 16. Certain conditions originating in the perinatal period	P00-P96	48
Chapter 17. Congenital malformations, deformations and chromosomal abnormalities	Q00-Q99	2320
Chapter 18. Symptoms, signs and abnormal clinical and laboratory findings, not elsewhere classified	R00-R99	7
Chapter 19. Injury, poisoning and certain other consequences of external causes	S00-T88	29
Chapter 20. External causes of morbidity	V00-Y99	0
Chapter 21. Factors influencing health status and contact with health services	Z00-Z99	5

*The ORPHAcodes were originally extracted from the 2018 version of the Orphanet nomenclature.
