# Supplementary figures and images for: The interoperability between the Spanish version of the International Classification of Diseases and ORPHAcodes: towards better identification of rare diseases
Source: Orphanet J Rare Dis. 2021 Mar 9;16:121. doi: 10.1186/s13023-021-01763-y (PMC7941896; doi:10.1186/s13023-021-01763-y)

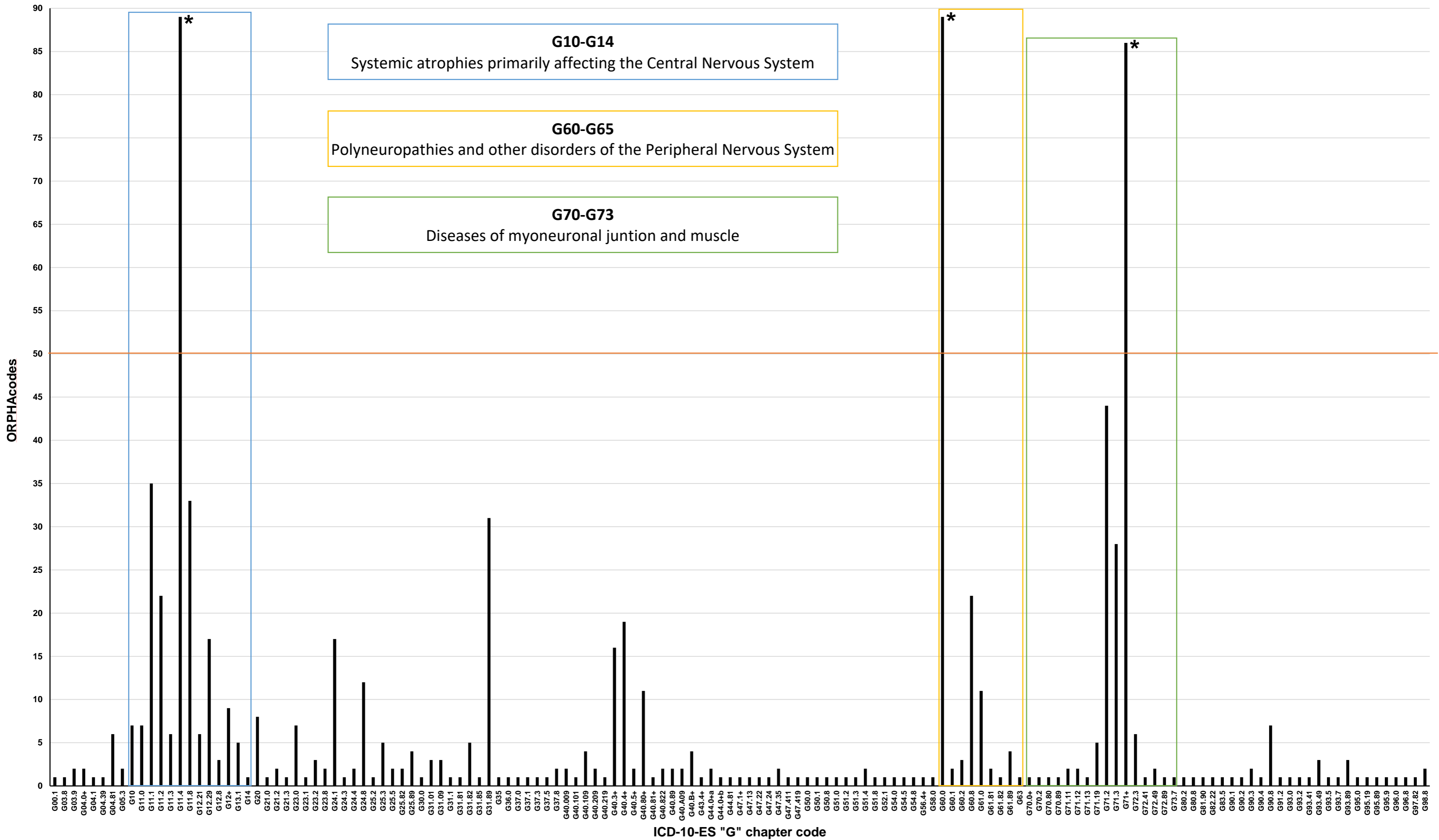

Supplementary Figure 1. Distribution of ORPHA codes per ICD-10-ES code of the G00-G99 chapter of the ICD-10-ES.

Supplement: Supplementary file 3 — Additional file 3: Fig. S1. Distribution of ORPHAcodes per ICD-10-ES code of the G00-G99 chapter of the ICD-10-ES [file 13023_2021_1763_MOESM3_ESM.pdf]

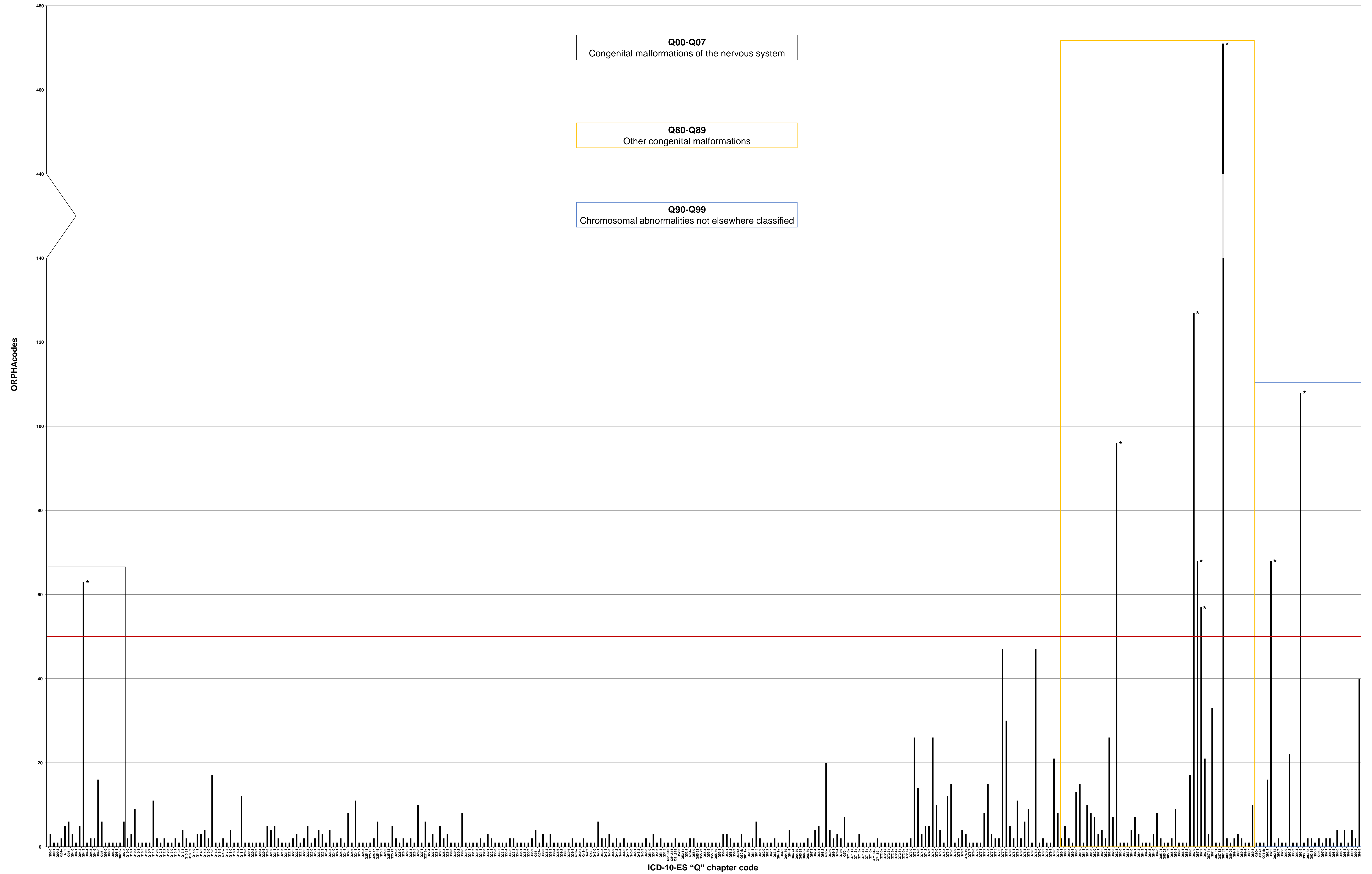

Supplementary Figure 2. Distribution of ORPHA codes per ICD-10-ES code of the Q00-Q99 chapter of the ICD-10-ES.

Supplement: Supplementary file 4 — Additional file 4: Fig. S2. Distribution of ORPHAcodes per ICD-10-ES code of the Q00-Q99 chapter of the ICD-10-ES [file 13023_2021_1763_MOESM4_ESM.pdf]
